# Stressful events throughout the life cycle and social inequalities in a cohort study

**DOI:** 10.1590/0102-311XEN052125

**Published:** 2025-12-01

**Authors:** Ingrid Medeiros Lessa, Bruna Gonçalves-Silva, Ana Maria Baptista Menezes, Fernando César Wehrmeister, Helen Gonçalves

**Affiliations:** 1 Programa de Pós-graduação em Epidemiologia, Universidade Federal de Pelotas, Pelotas, Brasil.

**Keywords:** Psychological Stress, Socioeconomic Factors, Demography, Social Inequalities, Birth Cohort, Estresse Psicológico, Fatores Socioeconômicos, Demografia, Desigualdades Sociais, Coorte de Nascimento, Estrés Psicológico, Factores Socioeconómicos, Demografía, Desigualdades Sociales, Cohorte de Nacimiento

## Abstract

Stressful events are avoidable and potentially traumatic situations that cause damage to physical and mental health. Research on this topic is generally carried out in contexts without significant social inequalities. We described the prevalence and inequality measures for exposure to different stressful events according to sex, skin color, family income, and schooling using data from five follow-ups of the 1993 Pelotas (Brazil) birth cohort. We evaluated stressful events until adolescence (< 18 years, n = 2,755), in adulthood (18-30 years, n = 1,752), and in both periods (n = 1,400). The characteristics of the analytical samples were approximated to baseline using inverse probability weighting. Simple and complex measures were used to measure inequalities (difference, ratio, SII, and CIX). Until adolescence, the most prevalent stressful events were parental separation (67.5% of men) and the death of a relative (66.1% of women). The death of a relative was the most prevalent stressful event for both sexes in adulthood (men: 65.8%; women: 63.2%) and in both periods (men: 44.1%; women: 44.2%). Regardless of the period of life, black, less educated, and poorer individuals were the most exposed to the majority of stressful events. Emotional neglect, incarceration, parental separation, and discrimination were among the most unequal exposures, with blacks, those with less schooling, and the poorest being most affected, women being more exposed to emotional neglect and discrimination, and men to the other exposures. Physical and mental health care programs should be developed to prevent such exposures and minimize their damage to health, especially in the most vulnerable groups.

## Abstract (pt)

Eventos estressantes são situações evitáveis ​​e potencialmente traumáticas que prejudicam a saúde física e mental. Pesquisas sobre esse tema geralmente são realizadas em contextos sem desigualdades sociais significativas. Descrevemos medidas de prevalência e de desigualdade para exposição a diferentes eventos estressantes de acordo com sexo, cor da pele, renda familiar e escolaridade, utilizando dados de cinco acompanhamentos da coorte de nascimentos de Pelotas (Brasil) de 1993. Avaliamos eventos estressantes até a adolescência (< 18 anos, n = 2.755), na idade adulta (18-30 anos, n = 1.752) e em ambos os contextos (n = 1.400). As características das amostras analíticas foram semelhantes à linha de base de acordo com a ponderação de probabilidade inversa. Utilizaram-se medidas simples e complexas para medir desigualdades (diferença, razão, índice de inclinação da desigualdade e índice de concentração). Até a adolescência, os eventos estressantes mais prevalentes foram separação dos pais (67,5% dos homens) e morte de um familiar (66,1% das mulheres). A morte de um familiar foi o evento estressante mais prevalente para ambos os sexos na idade adulta (homens: 65,8%; mulheres: 63,2%) e em ambos os períodos (homens: 44,1%; mulheres: 44,2%). Independentemente do período da vida, negros e indivíduos menos escolarizados e mais pobres foram os mais expostos à maioria dos eventos estressantes. Negligência emocional, encarceramento, separação dos pais e discriminação foram as exposições mais desiguais, com negros, aqueles com menor escolaridade e os mais pobres sendo os mais afetados, as mulheres sendo mais expostas à negligência emocional e à discriminação, e os homens às demais exposições. Programas de saúde física e mental devem ser desenvolvidos para prevenir tais exposições e minimizar os danos à saúde, especialmente nos grupos mais vulneráveis.

## Abstract (es)

Los acontecimientos estresantes son situaciones evitables y potencialmente traumáticas que causan daños a la salud física y mental. Las investigaciones sobre este tema se llevan a cabo generalmente en contextos sin desigualdades sociales significativas. Describimos las medidas de prevalencia y desigualdad en la exposición a diferentes eventos estresantes según el sexo, el color de la piel, los ingresos familiares y el nivel educativo, utilizando datos de cinco seguimientos de la cohorte de nacimientos de Pelotas (Brasil) de 1993. Evaluamos los eventos estresantes hasta la adolescencia (< 18 años, n = 2.755), en la edad adulta (18-30 años, n = 1.752) y en ambos períodos (n = 1.400). Las características de las muestras analíticas se aproximaron al valor de referencia mediante la ponderación inversa de probabilidad. Se utilizaron medidas simples y complejas para medir las desigualdades (diferencia, ratio, SII y CIX). Hasta la adolescencia, los acontecimientos estresantes más frecuentes fueron la separación de los padres (67,5% de los hombres) y la muerte de un familiar (66,1% de las mujeres). La muerte de un familiar fue el evento estresante más prevalente para ambos sexos en la edad adulta (hombres: 65,8%; mujeres: 63,2%) y en ambos periodos (hombres: 44,1%; mujeres: 44,2%). Independientemente del período de la vida, las personas negras, con menor nivel educativo y más pobres fueron las más expuestas a la mayoría de los acontecimientos estresantes. El abandono emocional, el encarcelamiento, la separación de los padres y la discriminación se encontraban entre las exposiciones más desiguales, siendo las personas negras, las que tenían menos estudios y las más pobres las más afectadas. También se demostró que las mujeres eran más expuestas al abandono emocional y a la discriminación, y los hombres a las demás exposiciones. Se deben desarrollar programas de atención de la salud física y mental para prevenir tales exposiciones y minimizar su daño a la salud, especialmente en los grupos más vulnerables.

## Introduction

Brazil has historically faced complex social, political, and economic challenges, including economic, racial, and gender inequalities ^1^ ^,^ ^2^. Different sectors can be directly or indirectly affected by inequalities, some of which are essential Social Determinants of Health, including access to sanitation, water, housing, employment, and education, among others ^3^. Areas with significant social inequalities tend to have worse health outcomes, higher rates of lethal violence (homicide and suicide), and greater exposure to situations such as psychological abuse/violence, neglect/abandonment, physical abuse/violence, and sexual abuse/violence, especially when committed against children, young people, women, and black individuals ^4^.

Stressful events are situations (which may vary by country or location) that are harmful and avoidable, experienced one or more times throughout life, whether at home or in other environments ^5^ ^,^ ^6^ ^,^ ^7^. Evidence demonstrates that experiencing or witnessing the occurrence of stressful events from childhood onwards can interfere with the development of individuals, affecting their well-being and contributing to the occurrence of health problems ^8^. Individuals exposed to these situations during childhood or later stages of life face a greater risk of developing mental disorders, chronic non-communicable diseases, and other illnesses, as well as having short- and long-term social and professional losses ^9^ ^,^ ^10^ ^,^ ^11^ ^,^ ^12^ ^,^ ^13^ ^,^ ^14^ ^,^ ^15^ ^,^ ^16^.

Similar to inequalities, stressful events represent a longstanding and critical global public health issue that impacts national economies both directly and indirectly, resulting in healthcare expenditures and investment losses ^17^ ^,^ ^18^ ^,^ ^19^ ^,^ ^20^ ^,^ ^21^ ^,^ ^22^. Although extensive research and public policies have been developed to address these problems ^5^ ^,^ ^7^ ^,^ ^23^ ^,^ ^24^, there are still significant gaps. In countries with great social inequality, such as Brazil, no studies have carried out a descriptive analysis of the socioeconomic and demographic profile of victims of stressful events in different life cycle periods ^25^ ^,^ ^26^. Filling this gap is necessary so that actions and public policies can prevent exposure and mitigate its consequences for the health of the most vulnerable groups ^27^, generating data and actions that engage with the Sustainable Development Goals (SDGs), including reducing inequalities, promoting quality education, ensuring good health and well-being, advancing gender equality, and pursuing justice peace ^28^.

Our objective was to describe the prevalence of exposure to different types of stressful events during adolescence and adulthood according to the demographic and socioeconomic characteristics of the participants in the 1993 Pelotas (Brazil) birth cohort. In addition, we measured inequalities in exposure to stressful events according to social markers.

## Materials and methods

### The original study and the sample

This study used data from a population-based cohort, which has been monitoring the health of its participants for 30 years ^29^. The 1993 Pelotas (Brazil) birth cohort is a prospective longitudinal study made up of 5,249 participants from the 5,265 live births in 1993 whose mothers lived in the urban area of the city ^29^ ^,^ ^30^. Pelotas is located in the far south of Rio Grande do Sul State and has 325,685 inhabitants. In 2022, the majority of the population was aged between 20 and 44; women accounted for 53.3% of the population, 76% of people were white, 24% were black, and 94% of the population lived in urban areas. In 2010, the municipality’s Human Development Index (HDI) was 0.739 ^31^.

Data collected during the perinatal period and in follow-ups at 11, 15, 18, 22, and 30 years of age on the participants in the 1993 cohort were used in the analyses ^29^ ^,^ ^32^. Information was collected through face-to-face interviews up to the age of 22, which were guided by general questionnaires applied by trained interviewers. In all follow-ups, the confidential questionnaires (made up of more sensitive questions, including those related to drug use, alcohol consumption, fights or violence, and sexual relations, among others) were self-administered. From the 30-year follow-up, data for both questionnaires were collected using online, self-administered questionnaires using the REDCap system (https://redcapbrasil.com.br/) ^33^.

Three analytical samples were used, which were made up of participants who had complete information at all follow-ups for all the stressful events investigated, regardless of whether they had been exposed or not: (1) until adolescence (< 18 years, n = 2,755); (2) adulthood (18-30 years, n = 1,752); and (3) both periods (from childhood to 30 years, n = 1,400).

The first analytical sample was derived from the follow-up of 18-year-olds, whose responses to the questions about stressful events were related to the last year. Positive responses to the questions asked at age 22, considering the period of recall of some moment in life, were taken into account in the composition of the second analytical sample, but only when participants reported having been exposed after age 17 or who tested positive for these questions in previous follow-ups and reported not having been exposed.

### Stressful events

In this study, the following types of stressful events were considered: child maltreatment (emotional neglect, physical and sexual abuse), death and parental separation, death of a relative or close person, serious financial difficulties, discrimination, relationship dissolution, incarceration, moving house against your will, community fear/insecurity, mother’s mental problems, domestic, physical and sexual violence, emotional neglect experienced in adulthood (Supplementary Material - Box S1; https://cadernos.ensp.fiocruz.br/static//arquivo/suppl-e00052125_8216.pdf).

These exposures were divided into three groups: (1) those experienced until adolescence (with information collected at 11, 15, and 18 years of age); (2) those experienced in adulthood (with information collected at 22 and 30 years of age); and (3) those experienced in both periods. Participants who tested positive for a specific type of stressful event until adolescence and adulthood were considered exposed in the third analytical sample. Those not exposed were participants who tested negative for the same kind of stressful event in at least one period.

It should be noted that in the 22-year follow-up, some questions were also asked about emotional neglect and domestic violence experienced up until adolescence. The recall period of the questions (shown in Supplementary Material - Box S1; https://cadernos.ensp.fiocruz.br/static//arquivo/suppl-e00052125_8216.pdf) varied between the last 6 months, the previous year, and lifetime.

Information on abuse and neglect experienced until adolescence (< 18 years of age) was collected using a questionnaire based on the translated and validated Portuguese version of the *Childhood Trauma Questionnaire* (CTQ) ^34^ ^,^ ^35^. In the definition of “physical abuse” in the 15-year follow-up, only those who answered that they had been beaten by their father, mother, stepfather, stepmother, grandfather, or grandmother were considered to have been exposed.

Mothers’ mental problems were assessed using the *Self-Reporting Questionnaire* (SRQ-20) ^36^, which was administered to the participants’ mothers at the 11-year follow-up by trained psychologists. According to Brazilian standardization, an SRQ-20 score of eight points or more was classified as positive for mental health problems ^37^.

Exposures to stressful events were assessed in different ways: (a) any type of stressful events (yes/no); (b) each type of stressful event (yes/no); (c) cumulative number of stressful events (categorized into 0, 1 to 2, 3 or more). For the types with more than one question in the questionnaire, participants who answered “yes” to at least one of the questions were considered exposed. Those who answered “yes” to at least one of the types were included in the cumulative number.

### Demographic and socioeconomic characteristics

Demographic characteristics included sex (male and female) and skin color, which were categorized as black and white. Yellow people and indigenous people were not included in the analysis due to their low prevalence in the overall sample (1.8% and 1.9%, respectively).

Sociodemographic variables included family income, categorized into quintiles (Q1 = poorest, Q2, Q3, Q4, and Q5 = richest), and schooling, measured in completed years of study (0-8, 9-11, and 12 or more). For the first analytical sample, data collected at the 18-year follow-up were used, while data collected at the 30-year follow-up were utilized for the second and third samples.

### Statistical analysis

Statistical analyses were performed using Stata software, version 15.0 (https://www.stata.com). Inverse probability weighting (IPW) was used to account for non-random missing data for the stressful events of interest in each analytical sample. We created indicators for each of the three analytical samples to flag missing data based on the stressor event scores. Observations lacking data for any type of stressor event were marked as missing. In each analytical sample, the weights were calculated using a logistic regression, where the missing indicator variable was used as the dependent variable, and the following variables collected in the perinatal period were used to adjust for selection bias: participant’s sex (male or female), family income (in minimum wages), maternal schooling (in years of completed schooling) and mother’s skin color (white, black or other). After calculating the weights, an adjustment was made for the missing observations, which received a weight of 0. The weighted data set was then defined for use in the descriptive analysis of the prevalence of exposure to stressful events.

The prevalence was described according to the demographic and socioeconomic characteristics collected in adolescence (in the case of the first analytical sample) and in adulthood - at the age of 30 (in the case of the second and third analytical samples) - using absolute and relative frequencies. The association between the variables was checked by bivariate analysis, using the chi-square test and the trend chi-square in the presence of ordinal variables.

To facilitate interpretation, the inequalities in exposure to stressful events according to sex/skin color/income/schooling will be presented graphically using the Equiplot (https://www.equidade.org/en/equiplot), which allows us to visualize both the prevalence and the inequality (represented by the distance between the points) of exposure within each group, and between them. Inequalities in exposure according to sex, skin color, and the extremes (poorest and richest, and less and more schooled) of the ordinal variables were measured using simple absolute (differences) and relative (ratios) measures of inequality. The slope index of inequality (SII) and the concentration index (CIX) were used as complex absolute and relative measures, respectively, to assess inequalities in exposure between categories of ordinal variables (household income and schooling level) ^38^.

In both measures (SII and CIX), positive values indicate that the indicator/concentration is higher among the more advantaged, negative values represent that the indicator/concentration is higher among the less advantaged, and “zero” means no inequality ^39^. The SII is an index derived from linear regression that measures absolute inequalities. It measures the slope of the regression line, calculated as the absolute difference between the predicted values for the most and least advantaged group in the socioeconomic distribution ^39^. The CIX is a relative measure, similar to the Gini coefficient, used to measure the concentration of the indicator in the socio-economic distribution. It varies between -1 and +1 and is based on the area between the concentration curve and the line of perfect equality, representing the relative inequality in the distribution of the indicator ^39^.

### Ethical issues

All the follow-ups of the 1993 cohort were approved by the Research Ethics Committee of the Faculty of Medicine of the Federal University of Pelotas. The approval protocol numbers for each follow-up were 4.06.01.095 (11 years), 158/07 (15 years), 05/2011 (18 years), 1.250.366 (22 years), and 5.911.994 (30 years).

## Results

Initially, 50% of the cohort sample was made up of women; approximately one-third was made up of black people, around 42% had a family income of between 1.1 and 3 minimum wages, almost 46% were the children of mothers with between 5 and 8 years of schooling, and only 8% were the children of mothers with 12 years or more of schooling. After weighting, the standardized differences between accompanied and unaccompanied were reduced, ranging from -0.008 to 0.002. The proportions of sociodemographic characteristics that make up the original cohort and each of the analytical samples are shown in Table 1.

**Table 1:** Demographic and socioeconomic characteristics in the perinatal period of the participants in the original the 1993 Pelotas (Brazil) birth cohort (n = 5,249), in the adolescence (n = 2,755) and adulthood samples (n = 1,752) and in both periods (n = 1,400).

Characteristics	Original sample perinatal		Participants included					
			Adolescence		Adulthood		Both periods	
	%	95%CI	%	95%CI	%	95%CI	%	95%CI
Sex								
Male	49.6	48.2; 50.9	49.6	47.7; 51.5	49.6	47.2; 52.0	49.7	47.0; 52.4
Female	50.4	49.0; 51.7	50.4	48.5; 52.3	50.4	48.0; 52.8	50.3	47.6; 53.0
Skin color *								
Black	33.5	32.1; 34.9	32.7	30.9; 34.5	34.0	31.8; 36.4	33.2	30.8; 35.8
White	66.5	65.0; 67.9	67.3	65.5; 69.0	66.0	63.6; 68.2	66.7	64.2; 69.8
Family income (minimum wages)								
≤ 1	18.8	17.7; 19.9	17.1	15.7; 18.6	17.3	15.6; 19.2	16.6	14.7; 18.7
1.1-3	41.8	40.4; 43.1	41.8	39.9; 43.7	41.8	39.4; 44.1	41.9	39.3; 44.6
3.1-6	23.4	22.3; 24.6	25.7	24.0; 27.4	25.6	23.6; 27.7	25.9	23.6; 28.3
≥ 6.1	15.9	14.9; 16.9	15.4	14.1; 16.9	15.3	13.7; 17.1	15.6	13.7; 17.6
Maternal schooling (years)								
0-4	28.0	26.8; 29.2	26.2	24.6; 27.9	27.4	25.3; 29.6	26.9	24.6; 29.4
5-8	46.2	44.9; 47.6	49.2	47.3; 51.0	47.1	44.7; 49.5	47.9	45.3; 50.6
9-11	17.6	16.6; 18.7	17.4	16.0; 18.8	17.9	16.2; 19.8	17.9	15.9; 20.0
12 or more	8.1	7.4; 8.9	7.2	6.3; 8.3	7.6	6.5; 8.9	7.2	6.0; 8.7

95%CI: 95% confidence interval.

* Variable with the highest number of missing (n = 4,164).

Exposure to any type of stressful events was highly prevalent (≥ 88.5%) in all periods of life. Until adolescence, the death of a relative/close person and parental separation were the most common stressful events. In adulthood and in both periods, death of a relative/close relative and fear/insecurity of the community were the most prevalent stressful events (Table 2).

**Table 2:** Prevalence of the types of stressful events experienced up to adolescence and adulthood and both periods. Follow-up data from 11 to 30 years of the 1993 Pelotas (Brazil) birth cohort.

Stressful event	Adolescence		Adulthood		Both periods	
	%	95%CI	%	95%CI	%	95%CI
Physical abuse/violence	56.9	55.0; 58.7	18.1	16.4; 20.1	11.1	9.5; 12.9
Sexual abuse/violence	1.4	1.0; 1.9	3.0	2.3; 3.9	0.1	0.0; 0.6
Emotional neglect	32.6	30.8; 34.4	41.1	38.7; 43.4	17.2	15.3; 19.3
Death of parents	10.0	8.9; 11.2	15.5	13.8; 17.3	1.0	0.6; 1.6
Parental divorce	64.3	62.5; 66.1	8.1	6.9; 9.5	3.9	3.0; 5.1
Death of a relative/close person	64.7	62.8; 66.5	64.5	62.2; 66.7	44.2	41.5; 46.8
Financial difficulties	22.2	20.7; 23.9	39.6	37.3; 42.0	10.9	9.4; 12.7
Discrimination	15.1	13.8; 16.5	30.9	28.7; 33.1	6.2	5.0; 7.6
Domestic violence	28.9	27.2; 30.6	0.7	0.4; 1.2	0.1	0.0; 0.6
Relationship dissolution	20.6	19.1; 22.2	31.4	29.3; 33.7	7.8	6.5; 9.4
Incarceration	4.2	3.5; 5.1	5.0	4.0; 6.2	0.0	-
Moving out against their will	5.9	5.1; 6.9	13.4	11.8; 15.1	1.2	0.7; 1.9
Community fear/insecurity	57.1	55.3; 59.0	58.3	55.9; 60.6	28.0	25.6; 30.5
Mother’s mental disorders	30.0	28.3; 31.8	-	-	-	-

95%CI: 95% confidence interval.

### Exposure to stressful events according to demographic characteristics

**Figure 1:**
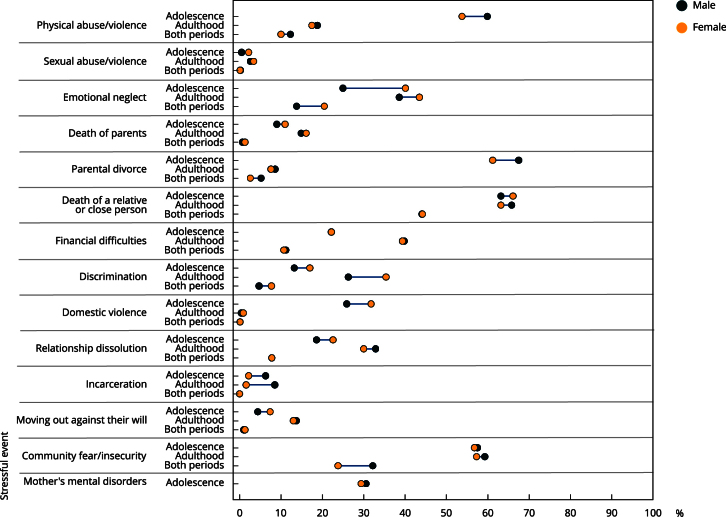
Prevalence of the types of stressful events experienced up to adolescence and adulthood and in both periods of life according to sex. Follow-up data from 11 to 30 years of the 1993 Pelotas (Brazil) birth cohort.

Until adolescence, the most prevalent stressful events for both sexes were the death of a relative/close person (63.2% male; 66.1% female) and parental separation (67.5% male; 61.2% female). In adulthood, the most prevalent were the death of a relative/close person (65.8% male; 63.2% female) and community fear/insecurity (59.3% male; 57.3% female). In both periods, the death of a relative/person continued to be the most prevalent (approximately 44% in both sexes) (Figure 1).

The absolute and relative measures indicated variations in inequalities between the sexes according to the type of stressful event and stage of life. Until adolescence, females were 15.1 percentage points (p.p.) more exposed to emotional neglect, and males were 190% more exposed to incarceration (Table 3). In adulthood, the most significant inequalities were related to discrimination (females were 9.1 p.p. more exposed) and imprisonment (males were 430% more exposed). In both periods of life, the greatest inequalities between sexes were observed in exposure to community fear/insecurity (men were 8.4 p.p. more exposed) and emotional neglect (women were 6.7 p.p. more exposed) (Table 3).

**Table 3:** Absolute and relative measures of inequalities for exposure to each type of stressful event experienced up to adolescence (n = 2,755) and adulthood (n = 1,752) and in both periods of life (n = 1,400) according to sex and skin color. Data from the 1993 Pelotas (Brazil) birth cohort.

Stressful event	Adolescence							
	Sex (male n = 1,341; female n = 1,364)				Skin color (black n = 854; white n = 1,757)			
	Difference * (p.p.)	95%CI	Ratio **	95%CI	Difference * (p.p.)	95%CI	Ratio **	95%CI
Physical abuse	6.1	2.0; 10.0	1.1	1.0; 1.2	12.8	8.9; 16.8	1.2	1.2; 1.3
Sexual abuse	-1.7	-2.5; -0.8	0.2	0.1; 0.5	0.5	-0.5; 1.4	1.4	0.7; 2.7
Emotional neglect	-15.1	-19.0; -12.0	0.6	0.5; 0.7	4.9	0.9; 8.7	1.1	1.0; 1.3
Death of parents	-2.0	-4.2; 0.3	0.8	0.6; 1.0	2.9	0.3; 5.4	1.3	1.0; 1.7
Parental divorce	6.3	2.7; 10.0	1.1	1.0; 1.2	12.7	9.0; 16.5	1.2	1.1; 1.3
Death of a relative/close person	-2.9	-6.5; 0.6	0.9	0.9; 1.0	5.7	1.8; 9.6	1.1	1.0; 1.2
Financial difficulties	0.0	-3.1; 3.2	1.0	0.9; 1.1	6.4	2.9; 9.9	1.3	1.1; 1.5
Discrimination	-3.8	-6.5; -1.1	0.8	0.6; 0.9	4.3	1.2; 7.3	1.3	1.1; 1.6
Domestic violence	-5.9	-9.2; -2.4	0.8	0.7; 0.9	7.0	3.1; 10.7	1.3	1.1; 1.4
Relationship dissolution	-4.0	-7.0; -1.0	0.8	0.7; 0.9	-0.3	-3.6; 2.9	1.0	0.8; 1.1
Incarceration	4.1	2.5; 5.6	2.9	1.9; 4.3	1.3	-0.4; 3.0	1.3	0.9; 1.9
Moving out against their will	-3.0	-4.8; -1.2	0.6	0.4; 0.8	1.2	-0.7; 3.1	1.2	0.8; 1.7
Community fear/insecurity	0.7	-2.9; 4.5	1.0	0.9; 1.1	-3.3	-7.3; 0.8	0.9	0.9; 1.0
Mother mental disorders	1.2	-2.2; 4.7	1.0	0.9; 1.2	7.5	3.6; 11.3	1.3	1.1; 1.4
	Adulthood							
	Sex (male n = 853; female n = 866)				Skin color (black n = 544; white n = 1,055)			
	Difference * (p.p.)	95%CI	Ratio **	95%CI	Difference * (p.p.)	95%CI	Ratio **	95%CI
Physical violence	1.3	-2.4; 4.8	1.1	0.9; 1.3	1.4	-2.6; 5.5	1.1	0.9; 1.3
Sexual violence	-0.8	-2.4; 0.8	0.8	0.4; 1.3	1.3	-0.7; 3.1	1.5	0.8; 2.5
Emotional neglect	-4.9	-9.6; -0.3	0.9	0.8; 1.0	7.6	2.4; 12.7	1.2	1.1; 1.3
Death of parents	-1.2	-4.7; 2.2	0.9	0.7; 1.1	1.3	-2.5; 5.1	1.1	0.8; 7.4
Parental divorce	1.0	-1.6; 3.5	1.1	0.8; 1.5	-1.3	-4.0; 1.5	0.8	0.6; 1.2
Death of a relative/close person	2.6	-2.0; 7.0	1.0	1.0; 1.1	10.0	5.1; 14.7	1.2	1.1; 1.2
Financial difficulties	0.4	-4.2; 5.0	1.0	0.9; 1.1	11.3	6.2; 16.4	1.3	1.2; 1.5
Discrimination	-9.1	-13.5; -4.8	0.7	0.6; 0.8	14.8	9.8; 19.6	1.6	1.4; 1.8
Domestic violence	-0.5	-1.3; 0.2	0.4	0.1; 1.4	0.5	-0.4; 1.5	2.0	0.6; 6.0
Relationship dissolution	2.9	-1.6; 7.2	1.1	0.9; 1.2	6.8	1.9; 11.7	1.2	1.1; 1.4
Incarceration	6.9	5.0; 9.0	5.3	3.0; 9.3	0.7	-1.6; 3.1	1.1	0.7; 1.8
Moving out against their will	0.7	-2.5; 3.9	1.0	0.8; 1.3	2.4	-1.2; 5.9	1.2	0.9; 1.5
Community fear/insecurity	2.0	-2.7; 6.7	1.0	0.9; 1.1	-5.1	-10.3; -0.1	0.9	0.8; 1.0
	Both periods							
	Sex (male n = 683; female n = 691)				Skin color (black n = 441; white n = 886)			
	Difference * (p.p.)	95%CI	Ratio **	95%CI	Difference * (p.p.)	95%CI	Ratio **	95%CI
Physical abuse/violence	2.3	-1.0; 5.6	1.2	0.9; 1.6	0.8	-2.8; 4.5	1.1	0.8; 1.5
Sexual abuse/violence	0.1	-0.4; 0.4	2.0	0.1; 16.1	0.1	-0.4; 0.6	2.0	0.1; 32.0
Emotional neglect	-6.7	-10.6; -2.7	0.7	0.5; 0.8	6.6	2.1; 11.2	1.4	1.1; 1.8
Death of parents	-0.6	-1.6; 0.5	0.5	0.2; 1.7	0.6	-0.7; 1.8	1.7	0.6; 5.1
Parental divorce	2.6	0.4; 4.5	2,0	1.1; 3.4	1.5	-0.9; 3.8	1.4	0.8; 2.4
Death of a relative/close person	-0.1	-5.3; 5.2	1.0	0.9; 1.1	12.3	6.5; 17.9	1.3	1.1; 1.5
Financial difficulties	0.5	-3.0; 3.7	1.0	0.8; 1.4	5.9	2.0; 9.6	1.6	1.2; 2.2
Discrimination	-3.0	-5.5; -0.4	0.6	0.4; 0.9	5.3	2.3; 8.4	2.3	1.5; 3.5
Domestic violence	0.0	-0.4; 0.4	1.0	0.0; 16.1	0.1	-0.4; 0.6	2.0	0.1; 32.0
Relationship dissolution	0.0	-0.3; 0.3	1.0	0.7; 1.4	2.4	-0.9; 5.5	1.3	0.9; 1.9
Incarceration	0.0	0.0; 0.0	-	-	0.0	0.0; 0.0	-	-
Moving out against their will	-0.3	-1.4; 0.8	0.8	0.3; 2.1	0.3	-0.9; 1.6	1.3	0.5; 3.7
Community fear/insecurity	8.4	3.7; 13.2	1.3	1.1; 1.6	-4.8	-9.8; 0.3	0.8	0.7; 1.0

95%CI: 95% confidence interval; p.p.: percentage points.

* Difference = male - female; black - white;

** Ratio = male / female; black / white.

**Figure 2:**
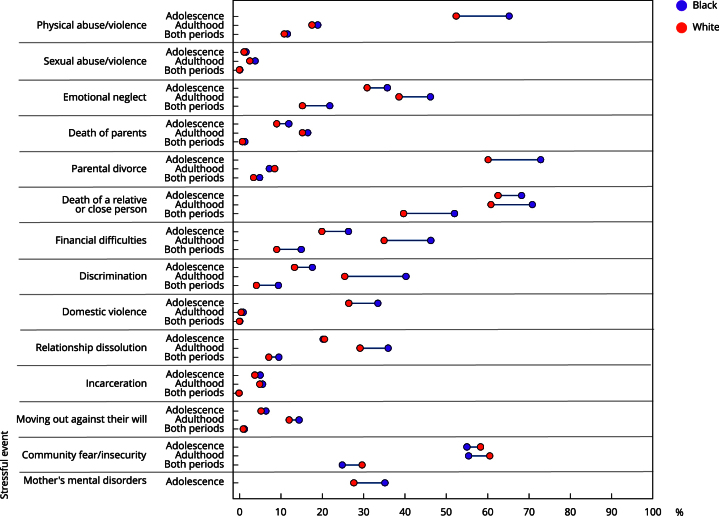
The prevalence of the types of stressful events experienced up to adolescence and adulthood and in both periods of life according to skin color. Follow-up data from 11 to 30 years of the 1993 Pelotas (Brazil) birth cohort.

With regard to skin color, until adolescence, the most prevalent stressful events were separation from parents (72.8% black; 60.1% white) and death of a relative/close person (68.2% black; 62.5% white). In adulthood, two stressful events also stood out as the most prevalent: death of a relative/close person (70.8% black; 60.8% white) and fear/insecurity in the community (55.4% black; 60.5% white). In both periods, the death of a relative was the most prevalent stressful event (52% black; 39.7% white), showing the disparities between skin color and exposure to stressful events over time (Figure 2).

The measures of absolute and relative inequalities revealed that regardless of the life stage, black individuals were exposed to more stressful events than white individuals. Until adolescence, black individuals were 12.8 p.p. more exposed to physical abuse and 12.7 p.p. more exposed to parental separation. In adulthood, black individuals were 14.8 p.p. more exposed to discrimination. In both periods, black individuals were 12.3 p.p. more exposed to the death of a relative/close person and 130% more exposed to discrimination (Table 3).

### Exposure to stressful events according to socioeconomic characteristics

**Figure 3:**
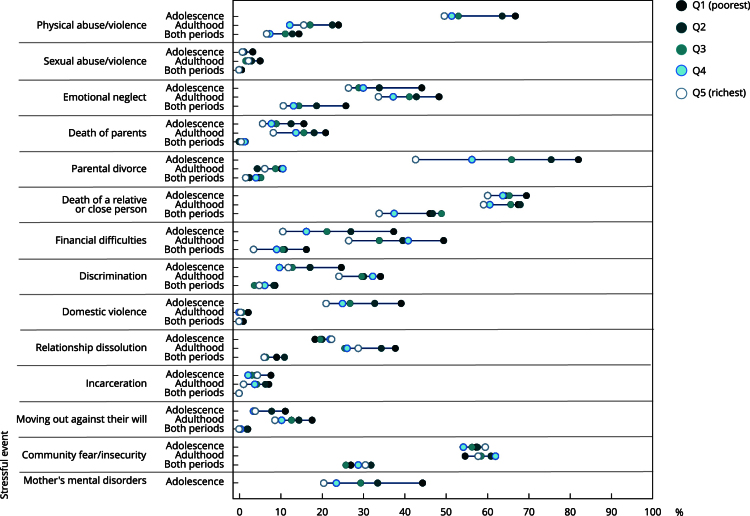
The prevalence of the types of stressful events experienced up to adolescence and adulthood and in both periods of life according to income. Follow-up data from 11 to 30 years of the 1993 Pelotas (Brazil) birth cohort.

Until adolescence, the most prevalent stressful events in the two poorest quintiles were separation from parents (82.3% Q1; 75.7% Q2) and death of a relative/close person (69.7% Q1; 64.5% Q2). In the richest quintiles, the death of a relative/close person was the most prevalent stressful event (64% Q4; 60.3% Q5), followed by parental separation in Q4 (56.5%) and community fear/insecurity in Q5 (59.7%). In adulthood, the most prevalent stressful events in all income quintiles were the death of a relative/close person (with the highest prevalence in Q2, at 68.2%) and community fear/insecurity (with the highest prevalence in Q4, at 62.2%). In both periods of life, the death of a relative/close person continued to be the most prevalent exposure in all income quintiles, and the highest prevalence was observed in Q3 (49.1%) (Figure 3).

The SII and CIX revealed that in all periods of life, the poor were more affected by most of the stressful events than the rich. The greatest inequalities observed, with the poor being the most exposed, relate to parental separation (46.2 p.p.) and sexual abuse (CIX = -0.3) - until adolescence; financial difficulties (21.4 p.p.) and domestic violence (CIX = -0.4) - adulthood; and emotional neglect and the death of a relative/close person (17.0 p.p. each) - both periods (Table 4).

**Table 4:** Simple and complex measures of inequality for each type of stressful event) experienced up to adolescence (n = 2,755) and adulthood (n = 1,752) and in both periods of life (n = 1,400) according to family income (in quintiles). Data from the 1993 Pelotas (Brazil) birth cohort.

Stressful event	Adolescence							
	SII (p.p.)	95%CI	CIX	95%CI	Difference * (p.p.)	95%CI	Ratio **	95%CI
Physical abuse	-22.2	-28.5; -16.0	-0.1	0.0; -0.1	17.2	11.4; 23.1	1.3	1.2; 1.5
Sexual abuse	-2.5	-4.3; -0.7	-0.3	-0.5; -0.1	2.5	0.7; 4.2	4.1	1.5; 13.0
Emotional neglect	-18.6	-24.7; -12.6	-0.1	-0.1; -0.1	17.8	12.1; 23.5	1.7	1.4; 2.0
Death of parents	-12.0	-16.1; -7.9	-0.2	-0.3; -0.1	10.0	6.3; 13.8	2.7	1.9; 4.1
Parental divorce	-46.2	-51.5; -40.8	-0.1	-0.1; -0.1	39.5	34.0; 44.7	1.9	1.7; 2.1
Death of a relative/close person	-9.3	-15.5; -3.1	0.0	0.0; 0.0	9.4	3.8; 15.3	1.1	1.0; 1.3
Financial difficulties	-31.0	-36.2; -25.7	-0.2	-0.3; -0.2	26.9	21.9; 31.9	3.5	2.7; 4.6
Discrimination	-15.8	-20.7; -10.8	-0.2	-0.2; -0.1	12.9	8.2; 17.5	2.1	1.6; 2.7
Domestic violence	-21.1	-26.9; -15.3	-0.1	-0.1; -0.1	18.2	12.8; 23.7	1.9	1.5; 2.3
Relationship dissolution	4.9	-0.4; 10.2	0.0	0.0; 0.1	-4.0	-8.8; 0.9	0.8	0.6; 1.0
Incarceration	-3.9	-7.0; -0.8	-0.2	-0.3; 0.0	3.3	0.5; 6.3	1.7	1.1; 2.9
Moving out against their will	-9.1	-12.7; -5.6	-0.2	-0.3; -0.2	7.3	3.9; 10.4	2.9	1.7; 4.5
Community fear/insecurity	0.5	-6.0; 7.0	0.0	0.0; 0.0	-2.2	-8.2; 3.7	1.0	0.9; 1.1
Mother mental disorders	-27.5	-33.3; -21.8	-0.1	-0.2; -0.1	24.0	18.5; 29.5	2.2	1.8; 2.6
	Adulthood							
	SII (p.p.)	95%CI	CIX	95%CI	Difference * (p.p.)	95%CI	Ratio **	95%CI
Physical violence	-13.2	-20.4; -5.9	-0.1	-0.1; 0.0	8.4	1.7; 15.1	1.5	1.1; 2.1
Sexual violence	-3.0	-6.5; 0.4	0.0	-0.2; 0.1	2.8	-0.5; 6.0	2.2	0.9; 5.2
Emotional neglect	-17.0	-25.8; -8.2	0.0	-0.1; 0.0	14.7	6.4; 22.7	1.4	1.2; 1.7
Death of parents	-14.6	-21.1; -8.1	-0.1	-0.2; 0.0	12.7	6.7; 18.4	2.5	1.6; 3.9
Parental divorce	- 0.8	-5.9; 4.2	0.0	-0.1; 0.1	4.0	-0.8; 8.4	1.6	0.9; 2.8
Death of a relative/close person	-11.9	-20.6; -3.2	0.0	0.0; 0.0	8.4	0.4; 16.4	1.1	1.0; 1.3
Financial difficulties	-21.4	-30.0; -12.8	0.0	-0.1; 0.0	23.0	15.2; 31.0	1.9	1.5; 2.3
Discrimination	-8.7	-17.0; -0.5	0.0	0.0; 0.0	10.1	2.7; 17.8	1.4	1.1; 1.9
Domestic violence	-2.0	-4.0; -0.1	-0.4	-0.7; 0.0	1.9	0.0; 3.8	7.3	0.8; 52.5
Relationship dissolution	-12.5	-21.1; -4.0	0.0	-0.1; 0.0	9.0	0.9; 16.7	1.3	1.0; 1.6
Incarceration	-7.4	-11.5; -3.3	-0.1	-0.2; 0.1	6.1	2.7; 9.4	6.5	2.0; 22.4
Moving out against their will	-10.9	-17.1; -4.7	0.0	-0.1; 0.0	9.0	3.5; 14.7	2.0	1.3; 3.3
Community fear/insecurity	-3.5	-5.5; 12.5	0.0	0.0; 0.0	-3.2	-11.6; 5.0	0.9	0.8; 1.1
	Both periods							
	SII (p.p.)	95%CI	CIX	95%CI	Difference * (p.p.)	95%CI	Ratio **	95%CI
Physical abuse/violence	-10.3	-16.7; -3.9	0.0	-0.1; 0.0	7.8	2.0; 13.7	2.2	1.2; 4.0
Sexual abuse/violence	0.0	-1.7; 0.5	-0.2	-1.3; 0.9	0.6	-0.5; 1.5	-	-
Emotional neglect	-17.0	-24.7; -9.3	-0.1	-0.1; 0.0	15.2	7.9; 22.6	2.4	1.6; 3.7
Death of parents	0.7	-0.7; 2.2	0.2	0.0; 0.4	-0.1	-1.2; 1.4	0.8	0.1; 18.7
Parental divorce	-2.4	-6.2; 1.4	0.0	-0.2; 0.2	3.2	-0.1; 6.9	3.0	0.9; 9.3
Death of a relative/close person	-17.0	-26.9; -7.1	0.0	0.0; 0.0	12.3	3.1; 21.6	1.4	1.1; 1.7
Financial difficulties	-13.4	-19.6; -7.2	0.0	-0.1; 0.1	12.8	7.1; 18.5	4.6	2.2; 10.0
Discrimination	-4.7	-9.9; 0.5	-0.1	-0.2; 0.0	3.8	-0.8; 8.7	1.8	0.9; 3.8
Domestic violence	-0.9	-2.1; 0.3	-0.9	-1.0; -0.7	1,0	-0.3; 2.4	-	-
Relationship dissolution	-5.4	-11.0; 0.2	-0.1	-0.2; 0.0	3.0	-2.0; 8.2	1.5	0.8; 3.0
Incarceration	-	0.0	0.0	-	-	-	-	-
Moving out against their will	-2.8	-5.1; -0.5	-0.1	-0.5; 0.3	2.1	0.1; 4.0	-	-
Community fear/insecurity	1.7	-7.7; 11.1	0.0	-0.1; 0.0	-3.5	-12.1; 5.0	0.9	0.6; 1.2

95%CI: 95% confidence interval; CIX: concentration index; SII: slope index of inequality; p.p.: percentage points.

* Difference = Q1 (poorest) - Q5 (richest);

** Ratio = Q1 (poorest) / Q5 (richest).

When analyzing economic inequalities by comparing only the extreme quintiles (poorest and richest) in the three periods, the absolute and relative measures indicated that the greatest economic inequalities occurred with the poorest being the most exposed, mainly to parental separation (39.5 p.p.), financial difficulties (26.9 p.p.) and sexual abuse (310%) - until adolescence; financial difficulties (23.0 p.p.), domestic violence (630%) and incarceration (550%) - adulthood; and emotional neglect (15.2 p.p.), financial difficulties (360%) and parental separation (200%) - both periods (Table 4).

**Figure 4:**
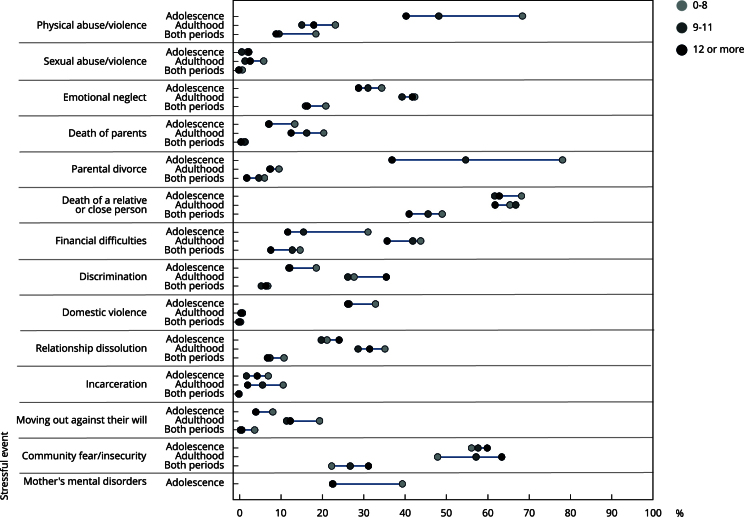
Prevalence of the types of stressful events experienced up to adolescence and adulthood and in both periods of life according to schooling. Follow-up data from 11 to 30 years of the 1993 Pelotas (Brazil) birth cohort.

Regarding education level, until adolescence, individuals with less schooling were more exposed to parental separation (78.2%), physical abuse (68.5%), and the death of a relative/close person (68.3%). Among those with more schooling, the most prevalent Stressful events were the death of a relative/close person (62.9%) and community fear/insecurity (60%). In adulthood, the death of a family member/close person was the most prevalent stressfull event in all categories of schooling and more prevalent among individuals with an intermediate level of schooling (66.9%). In addition, community fear/insecurity was also the most prevalent stressfull event among individuals with the highest level of schooling (63.5%). In both periods of life, the death of a relative/close person continued to be the most prevalent stressfull event, especially in the group with the lowest levels of education (49.1%) (Figure 4).

The SII and CIX revealed that until adolescence, the greatest inequalities occurred with less educated individuals being more exposed than those with higher levels of education, especially to parental separation (46.7 p.p.) and incarceration (CIX = -0.3). In adulthood, those with less schooling continued to be more exposed to incarceration (CIX = -0.3), and those with more schooling were 20.2 p.p. more exposed to community fear/insecurity. In both periods, the more schooling continued to be more exposed to community fear/insecurity (12.4 p.p.), and those with less schooling were more exposed to sexual abuse/violence (CIX = -0.8) (Table 5).

**Table 5:** Simple and complex measures of inequality for each type of stressful event experienced up to adolescence (n = 2,755) and adulthood (n = 1,752) and in both periods of life (n = 1,400) according to schooling (years). Data from the 1993 Pelotas (Brazil) birth cohort.

Stressful event	Adolescence							
	SII (p.p.)	95%CI	CIX	95%CI	Difference * (p.p.)	95%CI	Ratio **	95%CI
Physical abuse	-38.7	-45.1; -32.3	-0.1	-0.1; -0.1	28.1	18.2; 38.4	1.7	1.3; 2.2
Sexual abuse	-2.1	-4.0; -0.1	-0.2	-0.4; 0.0	-0.3	-3.0; 3.0	0.9	0.2; 4.1
Emotional neglect	-6.7	-13.5; 0.1	0.0	-0.1; 0.0	5.6	-3.8; 15.0	1.2	0.9; 1.6
Death of parents	-11.7	-16.3; -7.1	-0.2	-0.2; -0.1	6.3	0.7; 11.8	1.9	0.9; 3.9
Parental divorce	-46.7	-52.6; -40.9	-0.1	-0.1; -0.1	41.2	31.1; 50.9	2.1	1.6; 2.7
Death of a relative/close person	-11.8	-18.8; -4.9	0.0	0.0; 0.0	5.4	-4.5; 15.4	1.1	0.9; 1.3
Financial difficulties	-29.9	-35.7; -24.0	-0.2	-0.2; -0.1	19.4	13.0; 26.7	2.6	1.6; 4.8
Discrimination	-11.9	-17.2; -6.6	-0.1	-0.2; -0.1	6.6	-0.4; 13.3	1.5	0.9; 2.6
Domestic violence	-13.6	-20.2; -7.0	-0.1	-0.1; 0.0	6.7	-2.7; 15.6	1.2	0.9; 1.7
Relationship dissolution	-1.7	-7.7; 4.2	0.0	-0.1; 0.0	-2.9	-11.1; 6.4	0.9	0.6; 1.3
Incarceration	-9.8	-13.6; -6.0	-0.3	-0.4; -0.2	2.7	-1.2; 7.2	1.6	0.6; 4.6
Moving out against their will	-7.8	-11.5; -4.1	-0.2	-0.2; -0.1	4.1	-0.1; 8.3	2.0	0.7; 5.3
Community fear/insecurity	3.5	-3.7; 10.8	0.0	0.0; 0.0	-3.8	-13.8; 6.5	0.9	0.8; 1.1
Mother mental disorders	-31.0	-37.3; -24.7	-0.1	-0.2; -0.1	16.8	8.0; 25.6	1.7	1.2; 2.5
	Adulthood							
	SII (p.p.)	95%CI	CIX	95%CI	Difference * (p.p.)	95%CI	Ratio **	95%CI
Physical violence	-3.7	-10.7; 3.3	0.0	-0.1; 0.0	5.2	0.0; 10.6	1.3	1.0; 1.6
Sexual violence	-2.7	-6.1; 0.7	-0.1	-0.3; 0.1	3.3	0.6; 6.2	2.2	1.2; 4.1
Emotional neglect	1.0	-7.7; 9.7	0.0	0.0; 0.0	0.6	-5.8; 7.0	1.0	0.9; 1.2
Death of parents	-10.8	-17.1; -4.4	-0.1	-0.2; 0.0	7.9	2.9; 12.8	1.6	1.2; 2.2
Parental divorce	-2.5	-7.4; 2.4	0.0	-0.1; 0.1	2.2	-1.4; 6.0	1.3	0.9; 2.0
Death of a relative/close person	-7.0	-15.5; 1.5	0.0	0.0; 0.0	3.6	-2.6; 9.8	1.1	1.0; 1.2
Financial difficulties	-12.5	-21.0; -4.0	0.0	-0.1; 0.0	8.1	1.7; 14.4	1.2	1.0; 1.4
Discrimination	14.4	6.2; 22.6	0.1	0.0; 0.1	-7.8	-13.7; 1.9	0.8	0.6; 0.9
Domestic violence	-0.6	-2.0; 0.7	-0.2	-0.5; 0.1	0.4	-0.7; 1.5	1.8	0.4; 7.9
Relationship dissolution	-2.0	-10.4; 6.2	0.0	0.0; 0.0	3.7	-2.4; 9.9	1.1	0.9; 1.3
Incarceration	-11.5	-16.1; -7.0	-0.3	-0.4; -0.2	8.6	5.1; 12.1	5.1	2.9; 9.2
Moving out against their will	-6.8	-13.1; -0.5	-0.1	-0.1; 0.0	7.1	2.2; 12.0	1.6	1.2; 2.1
Community fear/insecurity	20.2	11.8; 28.6	0.0	0.0; 0.1	-15.5	-21.9; -9.1	0.7	0.6; 0.8
	Both periods							
	SII (p.p.)	95%CI	CIX	95%CI	Difference * (p.p.)	95%CI	Ratio **	95%CI
Physical abuse/violence	-10.4	-17.0; -3.8	-0.1	-0.2; 0.0	9.6	4.2; 14.8	2.1	1.4; 2.9
Sexual abuse/violence	-0.8	-2.0; 0.4	-0.8	-0.9; -0.7	0.8	-0.3; 1.9	-	-
Emotional neglect	-4.4	-12.0; 3.0	0.0	-0.1; 0.0	4.5	-1.1; 10.4	1.3	0.9; 1.7
Death of parents	-1.6	-3.6; 0.4	-0.4	-0.6; 0.0	1.0	-0.5; 2.7	3.0	0.7; 14.4
Parental divorce	-6.6	-10.6; -2.5	-0.2	-0.4; 0.0	4.3	1.2; 7.5	3.3	1.6; 6.8
Death of a relative/close person	-11.4	-21.1; -1.7	0.0	-0.1; 0.0	8.0	0.7; 15.2	1.2	1.0; 1.4
Financial difficulties	-10.7	-16.8; -4.6	-0.1	-0.2; 0.0	7.1	2.3; 12.0	1.9	1.3; 2.9
Discrimination	0.2	-4.7; 5.0	0.0	-0.1; 0.1	0.5	-3.2; 4.2	1.1	0.6; 1.8
Domestic violence	-0.5	-1.3; 0.3	-0.6	-0.8; -0.5	0.4	-0.4; 1.1	-	-
Relationship dissolution	-4.5	-10.1; 1.0	-0.1	-0.2; 0.0	4.0	-0.2; 8.4	1.6	1.0; 2.5
Incarceration	0.0	-	-	-	0.0	0.0; 0.0	-	-
Moving out against their will	-3.1	-6.0; -0.3	-0.4	-0.7; 0.0	3.1	0.5; 5.2	5.4	1.7; 17.7
Community fear/insecurity	12.4	3.5; 21.3	0.1	0.0; 0.1	-8.9	-15.3; -2.7	0.7	0.5; 0.9

95%CI: 95% confidence interval; CIX: concentration index; SII: slope index of inequality; p.p.: percentage points.

* Difference = (0-8) - (12 or more);

** Ratio = (0-8) / (12 or more).

When comparing only the less and more educated extremes, until adolescence, the less educated were more exposed to most of the stressful events, with parental separation (41.2 p.p.) and financial difficulties (160% greater exposure) being the stressful events with the greatest inequality of exposure. In adulthood, those with less schooling were 410% more exposed to incarceration, and those with more schooling were 15.5 p.p. more exposed to community fear/insecurity. In both periods of life, those with less schooling were more exposed to physical abuse/violence (a difference of 9.6 p.p.) and moved house against their will (440% more exposed) (Table 5).

### Cumulative exposure to different types of stressful events

Table 6 presents the prevalence of cumulative exposure to different types of stressful events. Until adolescence, regardless of sociodemographic characteristics, the highest prevalence rates observed were for exposure to three or more types. The most exposed groups included black individuals (83.7%), those from poorer families (87.8%), and individuals with lower schooling (87.5%).

**Table 6:** Cumulative exposure to different types of stressful events experienced up to adolescence (n = 2,755) and adulthood (n = 1,752) and in both periods of life (n = 1,400). Data from the 1993 Pelotas (Brazil) birth cohort.

Characteristics	Adolescence					
	0		1-2		3 or more	
	%	95%CI	%	95%CI	%	95%CI
Sex						
Male	1.5	1.0; 2.4	21.2	19.0; 23.6	77.2	74.8; 79.5
Female	1.5	1.0; 2.3	22.0	20.0; 24.2	76.4	74.2; 78.5
Skin color						
Black	1.5	0.9; 2.6	14.8	12.6; 17.3	83.7	81.1; 86.0
White	1.6	1.1; 2.3	24.9	22.9; 27.0	73.5	71.4; 75.6
Family income (quintiles)						
1st (poorest)	0.4	0.1; 1.7	11.8	9.2; 14.9	87.8	84.6; 90.4
2nd	0.9	0.4; 2.2	15.0	12.2; 18.3	84.1	80.7; 87.0
3rd	1.4	0.7; 2.8	20.5	17.4; 24.0	78.1	74.5; 81.3
4th	2.0	1.1; 3.6	26.8	23.2; 30.6	71.2	67.3; 74.9
5th (wealthy)	2.8	1.7; 4.6	33.0	29.1; 37.1	64.2	60.0; 68.2
Schooling (years)						
0-8	0.8	0.4; 1.5	11.7	9.9; 13.7	87.5	85.5; 89.4
9-11	2.2	1.6; 3.1	29.0	26.7; 31.4	68.8	66.3; 71.1
12 or more	0.8	0.1; 5.7	35.2	26.4; 45.2	63.9	54.0; 72.8
	Adulthood					
	0		1-2		3 or more	
	%	95%CI	%	95%CI	%	95%CI
Sex						
Male	4.8	3.5; 6.6	32.8	29.6; 36.3	62.3	58.8; 65.7
Female	4.9	3.7; 6.4	33.0	30.1; 36.1	62.1	58.9; 65.1
Skin color						
Black	3.9	2.5; 5.8	27.6	24.0; 31.5	68.5	64.6; 72.3
White	5.2	4.0; 6.8	36.3	33.4; 39.3	58.5	55.4; 61.4
Family income (quintiles)						
1st (poorest)	3.9	2.2; 6.9	26.1	21.2; 31.8	70.0	64.2; 75.2
2nd	4.0	2.2; 7.2	31.1	25.8; 37.0	64.9	58.9; 70.4
3rd	6.3	3.9; 10.0	31.4	26.2; 37.1	62.3	56.4; 67.8
4th	2.7	1.4; 5.4	37.6	32.1; 43.4	59.7	53.9; 65.2
5th (wealthy)	7.0	4.5; 10.6	42.4	36.7; 48.2	50.6	44.8; 56.4
Schooling (years)						
0-8	6.3	4.0; 9.6	30.8	25.9; 36.2	63.0	57.4; 68.2
9-11	5.9	4.3; 8.1	32.7	29.0; 36.6	61.4	57.4; 65.3
12 or more	3.4	2.3; 4.9	34.4	31.1; 37.8	62.2	58.8; 65.6
	Both periods					
	0		1-2		3 or more	
	%	95%CI	%	95%CI	%	95%CI
Sex						
Male	26.9	23.4; 30.6	57.5	53.5; 61.4	15.6	12.9; 18.8
Female	27.7	24.6; 31.0	57.0	53.5; 60.5	15.3	12.9; 18.0
Skin color						
Black	22.7	19.1; 26.7	57.0	52.4; 61.4	20.3	16.9; 24.3
White	29.9	26.9; 33.1	56.6	53.2; 59.9	13.5	11.4; 16.0
Family income (quintiles)						
1st (poorest)	20.7	15.7; 26.9	58.5	51.5; 65.2	20.7	15.6; 26.9
2nd	24.6	19.3; 30.8	56.8	50.1; 63.3	18.6	13.9; 24.4
3rd	25.0	19.8; 31.0	61.7	55.2; 67.8	13.3	9.5; 18.4
4th	32.2	26.4; 38.6	56.7	50.2; 63.1	11.0	7.5; 15.9
5th (wealthy)	34.8	28.8; 41.2	57.2	50.7; 63.5	8.0	5.1; 12.4
Schooling (years)						
0-8	27.5	22.2; 33.6	48.6	42.4; 55.0	23.8	18.9; 29.6
9-11	26.2	22.4; 30.3	58.8	54.3; 63.1	15.1	12.1; 18.5
12 or more	28.1	24.7; 31.8	59.5	55.6; 63.3	12.3	9.9; 15.2

95%CI: 95% confidence interval.

In adulthood, prevalence rates were lower, but the pattern of exposure was similar to that of adolescence: black individuals (68.5%) and those from low-income families (70%) continued to be the most exposed to three or more types of stressful events.

Although it did not show the highest prevalence, the highest level (three or more) of cumulative exposure to stressful events experienced in both periods of life showed considerable differences between categories: black individuals were 20.3% exposed, while white individuals were 13.5%; those belonging to the poorest quintile were 20.7% exposed, while those belonging to the richest quintile were only 8%; and those with less schooling were 23.8% exposed, while those with more schooling were 12.3% exposed.

## Discussion

Our results highlight significant disparities in exposure to stressful events based on sociodemographic characteristics. Men and women are affected differently, depending on the type of stressfull event experienced and the stage of life, and the social roles historically assigned to men and women in cultures where patriarchy is still predominant, influence the way both groups are exposed and respond to stressors. While evidence shows that men and women are exposed to and impacted by stressful events in distinct ways, longitudinal studies analyzing these exposures with sex stratification remain scarce ^40^ ^,^ ^41^.

Black individuals, those with lower income and education, were most exposed to the majority of stressful events, regardless of life stage. We hypothesize that these findings are linked to economic inequality and structural racism. Greater social vulnerability among these groups increases their susceptibility to violence, loss, and health risks due to socioeconomic, cultural, and systemic challenges ^42^ ^,^ ^43^ ^,^ ^44^.

In Brazil, structural inequalities based on gender/sex, race/skin color, and social class marginalize women, black individuals, and low-income populations, restricting opportunities and perpetuating vulnerability ^45^. These systemic dynamics amplify inequalities in exposure to stressful events, including financial difficulties, emotional neglect, discrimination, domestic violence, sexual abuse, and incarceration, as identified in this study. In this context, we highlight the importance of developing initiatives such as The Lancet Commission on global mental health and sustainable development ^46^, to achieve the SDGs, broadening the concept of health, reducing the avoidable suffering of victims and working to develop human potential, taking into account the social determinants of health and the critical periods of human development - childhood and adolescence ^47^.

Regarding exposure during adolescence, our findings confirm that women face a greater diversity of stressful events than men, reflecting gender/sex inequalities rooted in childhood ^48^. These disparities are made up of socioeconomic and racial factors ^49^ ^,^ ^50^: poor women (both white and black) often sacrifice personal development to assume domestic and caregiving responsibilities ^51^ ^,^ ^52^. Excessive protection in childhood, combined with society’s discouragement to seek help, decreases women’s ability to deal with adversity throughout their lives, increasing their vulnerability to emotional neglect, discrimination, and sexual abuse ^48^ ^,^ ^53^ ^,^ ^54^ ^,^ ^55^. In contrast, Brazilian men are taught from childhood to normalize aggression as a form of defense and social advancement ^56^, which may explain their higher prevalence of physical abuse exposure until adolescence and incarceration in adolescence/adulthood.

While individuals respond differently to stressful events exposure, such events can trigger prolonged effects or be mitigated by resilience ^57^ ^,^ ^58^. Studies show that stressful events exposure is not independent ^59^; exposure to one type often leads to others, and cumulative exposure is positively associated with developing various diseases ^13^ ^,^ ^16^.

In line with the Global Mental Health concept ^60^, our results provide evidence to inform multidimensional practices and policies - integrating social, cultural, and political perspectives - to tackle global health issues, particularly in contexts with socioeconomic inequalities, and to mitigate harm from pre-adulthood stressful events exposure. Unlike other studies that reported a lower prevalence of cumulative exposure to three or more stressful events ^61^, our findings - which investigated a wider range of stressors, including community-level events - identified a higher prevalence of cumulative exposures to three or more stressful events through adolescence and adulthood, and to one or two stressful events in both periods.

Methodological limitations include underreporting of sensitive events (e.g., sexual violence) due to self-reporting tools and perpetrator proximity. Recall bias (e.g., reporting over six months, one year, or lifetime) may lead to underestimation, though shorter timeframes risk omitting past experiences. Information bias, particularly regarding family income, and incomplete data on variables such as maternal mental health (available only up to age 11) also limit the analysis. Selection bias and missing data present potential limitations in studies based on cohort data with multiple follow-ups over time. However, to minimize this last limitation, we employed IPW to align the analytical samples with the baseline characteristics of the cohort, which we highlight as a key strength of the study.

We also highlight other strengths: using data from a highly recognized birth cohort, which allows for generalization to similar social contexts, and contributes to the development of strategies and programs to combat stressful events or mitigate the damage caused by these exposures, focusing on the most vulnerable groups; the longitudinal assessment of various stressful events which improves understanding of critical periods of exposure in all social groups and highlights disparities in life-stage vulnerabilities; and the integrated analysis of prevalence and inequalities, with a focus on sociodemographic markers, which helps to identify populations at risk.

## Conclusion

To our knowledge, this is the first study to examine stressful events exposure across life stages in Brazil. Our results described the profile of the groups most exposed to stressful events (women up to adolescence, men in adulthood, and black, poor and less educated people in both periods of life) and the magnitude of the inequalities in exposure. We believe that our results can support the development of evidence-informed policies, prioritizing high-risk groups and contributing to an assertive response to stressful events. Given the importance of this issue for public health, we recommend including it in global health agendas, such as the University of Washington’s Global Burden of Disease, and implementing programs supporting both physical and mental health in schools, especially in low-income areas that experience a predominance of racial and gender discrimination. Further research should explore the intensity of exposure and sociodemographic intersectionality.

## Data Availability

The research data are available upon request to the corresponding author.
