# Supplementary material for: Stressful events throughout the life cycle and social inequalities in a cohort study
Source: Cad Saude Publica. 2025 Dec 1;41(11):e00052125. doi: 10.1590/0102-311XEN052125 (PMC12688210; doi:10.1590/0102-311XEN052125)
Supplement: Supplementary Material [file 1678-4464-csp-41-11-EN052125-s.pdf]

SUPPLEMENTARY MATERIAL

Box S1 Questions about stressful events at the respective ages followed up (11, 15, 18, 22, and 30 years), 1993 Pelotas (Brazil) birth cohort.

**Questions**	**Ages**					**Variable**
	**11**	**15**	**18**	**22**	**30**	**Type of stressful events**
How many times have you been spanked by your parents in the last 6 months?	x	x				**Physical abuse**
Does anyone ever hit you? Who?		x				
Has an adult of your family or someone who was looking after you hit you in a way that left you hurt or bruised?		x				
Since last year, have you ever been caught by your father or mother?			x			
In the past twelve months, how many times has someone in your family hit you, pushed you, kicked you, or physically assaulted you without a weapon?				x		**Physical violence**
Has anyone in your family ever hit you, pushed you, kicked you, or physically assaulted you WITHOUT A WEAPON? How old were you when this last happened?					x	
In the past twelve months, how many times has someone who is not a member of your family hit you, pushed you, kicked you, or physically assaulted you without a weapon?				x		
Has anyone who is not a member of your family ever hit you, pushed you, kicked you, or physically assaulted you WITHOUT A WEAPON? Who has done this to you? How old were you when this last happened?					x	
Has anyone ever tried to do sexual things to you against your will, threatening or hurting you?		x				**Sexual abuse**
In the last twelve months, how many times has someone in your family grabbed you, touched or assaulted your intimate sexual parts against your will?				x		**Sexual violence**
Has anyone in your family ever grabbed, touched or assaulted your intimate sexual parts against your will? How old were you were you when this last happened?					x	
In the last twelve months, how many times has someone who is not a member of your family grabbed, touched or assaulted your intimate sexual parts against your will? your will?				x		
Has anyone outside your family ever grabbed, touched or assaulted your intimate sexual parts against your will? Who has done this to you? How old were you when it last time?					x	
Have you ever thought or felt that someone in your family hates you?		x		x**		**Emotional neglect**
Have you ever thought or felt that your parents did not want you to have been born?		x		x**		
In the last year, have you felt or been very lonely, without support from your family and the majority of your friends?				x	x	
In the last year, have you lost the friendship of people you cared about?				x	x	
Is <name>'s natural mother/father still alive?*	x	x				**Death of parents**
Are your mother/father still alive or has she/he died?			x	x	x	
Do you think that your parents' separation has affected you in any way?	x	x				**Parental separation**
Do you think your parents' separation was beneficial for you in any way?	x	x				
Are your parents separated?			x	x	x	
Since last year, have any of your close relatives died?	x		x	x		**Death of a relative or close person**
Since last year, has anyone close to you not been related to you died?			x	x	x	
Since last year, has your family had money problems that have hurt you a lot?	x					**Financial difficulties**
Since last year, have you had more serious money problems than usual?			x	x	x	
Since last year, have you felt discriminated against or disadvantaged because of your color or race?	x			x	x	**Discrimination**
Since last year, have you felt discriminated against or disadvantaged because of your religion or cult?	x			x	x	
Since last year, have you felt discriminated against or disadvantaged because you are poor or rich?	x			x	x	
Since last year, have you felt discriminated against or disadvantaged because of illness or physical disability?	x			x	x	
Since last year, have you felt discriminated against or disadvantaged because of your sexual choice or preference?				x	x	
Since last year, have you felt discriminated against or disadvantaged because of being a man or a woman?				x	X	
Since last year, have you felt discriminated against or disadvantaged because of other things?	x					
Has there ever been fights with physical assault in your household between adults or has		x		x**		**Domestic**

an adult ever assaulted a child or adolescent?						
Has someone in your family stolen something from you, using violence or threats? Who has ever done this to you? How old were you when it last happened?					x	**violence**
Since last year, have you ended a steady relationship or marriage?			x	x	x	**Relationship dissolution**
Have you ever been in an institution for minors? How old were you when you were in the institution?		x	x			**Incarceration**
Have you ever been arrested or detained?			x	x	x	
Since last year, have you had to move house against your will?			x	x	x	**Moving house against your will**
Since last year, have you been forced to move to a new neighborhood or city? How has this affected you?				x	x	
Have you ever felt afraid of living in your neighborhood?	x	x				**Community fear/insecurity**
Have you ever felt fear or insecurity in your neighborhood?			x	x		
Have you ever been mugged?			x	x		
Have you been mugged since last year?				x		
Have you been mugged since you were 20?					x	
Self-Reporting Questionnaire (SRQ-20)	x					**Mother mental problems**

* Questions asked of the participant's guardian.

** Questions about childhood and adolescence.
